# Pancreatic Stone Protein in Burns: Clinical Value of Bedside Testing—A Prospective Pilot Study

**DOI:** 10.3390/diagnostics16132129

**Published:** 2026-07-07

**Authors:** Moritz Billner, Philipp von Imhoff, Konrad Karcz, Vadym Burchak, Maximilian C. Stumpfe, Celena A. Soergel, Denis Ehrl

**Affiliations:** 1 Burn Unit, Department of Plastic, Reconstructive and Hand Surgery, Klinikum Nuremberg Hospital, Paracelsus Medical University, Breslauer Str. 201, 90471 Nuremberg, Germany; 2 Division of Hand, Plastic and Aesthetic Surgery University Hospital, Ludwig-Maximilians-Universität Munich, Marchioninistraße 15, 81377 Munich, Germany

**Keywords:** pancreatic stone protein, burns, burn intensive care, sepsis biomarkers, nosocomial infections

## Abstract

**Background:** Early detection of severe infections in burn patients is difficult due to confounding sterile inflammation. Previous research has shown that Pancreatic Stone Protein (PSP) is less affected by trauma and surgery. Therefore, this study investigated whether longitudinal PSP trends can distinguish sterile post-burn inflammation from clinically relevant infections and indicate response to antimicrobial therapy. **Methods:** This prospective pilot cohort study included 10 consecutive adult patients with moderate to severe burn injuries admitted to a specialized burn intensive care unit. PSP levels were measured using bedside testing (abioSCOPE^®^) daily over a 14-day observation period. Clinical parameters, burn severity as assessed by the Abbreviated Burn Severity Index (ABSI), and the occurrence of severe infectious complications, including pneumonia and bacteremia, were systematically recorded. PSP measurements were not used to guide clinical decision-making. **Results:** Patients who developed severe infectious complications (pneumonia and/or bacteremia; mean ABSI 8.5) showed a consistent and characteristic increase in PSP levels (>350 ng/mL) over time, with elevations preceding the clinical diagnosis of infection (24–120 h). In contrast, patients without pneumonia or bacteremia (mean ABSI 6) exhibited low and stable PSP (<150 ng/mL) concentrations throughout the observation period, despite the presence of burn-related injury and the expected sterile inflammatory response. **Conclusions:** In this exploratory cohort study distinct PSP trajectory patterns, with persistently low levels in non-infected patients and rising levels preceding clinically diagnosed infection in several cases, were observed. These preliminary findings suggest that longitudinal PSP monitoring may provide potential utility for infection surveillance in burn ICU patients. However, due to the exploratory design and very limited sample size, the findings should be interpreted cautiously and require validation in larger prospective multicenter studies before conclusions regarding clinical decision-making or patient outcomes can be drawn.

## 1. Introduction

Severe thermal injuries can lead to a profound and sustained systemic inflammatory response. This process is characterized by cytokine release, endothelial dysfunction, metabolic disorders and immune dysregulation, which often lead to persistently elevated inflammatory biomarkers independent of infection in severely burned patients [1,2]. In addition to this persistent inflammatory activation, severely burned patients frequently undergo repeated surgical or enzymatic debridement procedures and prolonged intensive care treatment, both of which may further amplify systemic inflammatory responses. Mechanical ventilation, invasive devices, and extensive wound surfaces further increase susceptibility to nosocomial infections and complicate infection surveillance in burn intensive care patients. Consequently, distinguishing infection from sterile post-burn inflammation remains particularly challenging in this patient population [3].

In extensive burns, commonly used inflammatory biomarkers such as C-reactive protein (CRP) are frequently elevated even in the absence of infection [4]. Although biomarkers such as procalcitonin (PCT) and interleukin-6 (IL-6) have demonstrated potentially improved reliability compared with conventional markers, their diagnostic interpretation is confounded by postoperative inflammatory responses and pathogen-specific variability [5,6,7,8]. At the same time, unnecessary or prolonged empirical antimicrobial therapy may contribute to antimicrobial resistance and drug-related adverse events. Therefore, biomarkers capable of more reliably differentiating infectious from non-infectious inflammatory responses may support not only earlier diagnosis of sepsis but also more targeted antimicrobial stewardship strategies in burn intensive care medicine. However, burn specific evidence remains limited.

Additionally, clinical scoring systems, including the systemic inflammatory response syndrome (SIRS) criteria and the National Early Warning Score 2 (NEWS2), are similarly limited in burn populations. Physiological responses to burn trauma and surgery often fulfill score thresholds independent of infection, resulting in low specificity for sepsis detection [3].

Previous research has shown that an acute phase protein, pancreatic stone protein (PSP), may represent a promising biomarker in critically ill patients with sepsis and systemic infections. The PSP is a secretory protein produced by pancreatic acinar cells and released in response to systemic stress and infection [9,10]. Previous studies in critically ill patients observed rising PSP concentrations in association with sepsis, often earlier than conventional inflammatory markers [11]. In addition, PSP expression appears to be less influenced by trauma severity or surgical interventions in the further course of treatment, indicating a potential relative independence from sterile inflammatory responses [12,13]. PSP concentrations have also been reported to correlate with disease severity, organ dysfunction, and mortality in septic conditions [13]. Beyond isolated biomarker concentrations, PSP may also be suitable for longitudinal assessment. In this context, Klein et al. reported promising observations regarding PSP dynamics in burn patients by laboratory testing of the PSP [12,13].

However, longitudinal bedside monitoring of PSP trajectories in severely burned intensive care patients remains insufficiently investigated.

Therefore, the present study was designed as an exploratory prospective pilot investigation to evaluate longitudinal bedside PSP trajectories in severely burned intensive care patients. We hypothesized that PSP concentrations would demonstrate relatively stable temporal behavior during sterile post-burn inflammation while increasing in association with clinically relevant infectious complications. In addition, PSP dynamics were descriptively compared with established inflammatory biomarkers and clinical scoring systems during the early intensive care course.

## 2. Materials and Methods

This prospective pilot cohort study was conducted at a certificated burn center in Bavaria, Germany. The period of our study spanned from March to September 2025. The study was conducted in accordance with the Declaration of Helsinki and has been approved by the local ethics committee (FMS_FP_023.25-I-5).

Inclusion criteria were admission to the burn intensive care unit, affected total body surface area (TBSA) ≥ 10%, and age ≥ 18 years. Exclusion criteria were expected death within 24 h of admission and known acute or chronic pancreatitis or pancreatic carcinoma. Written informed consent was obtained from the patient or a legal representative prior to enrollment.

All patients received standardized burn intensive care treatment according to institutional protocols, including fluid resuscitation, wound care management, surgical debridement where indicated and infection surveillance. Antimicrobial treatment decisions were based on clinical evaluation, microbiological findings, and conventional laboratory parameters.

Our initial sample consisted of fifteen patients. Five patients were excluded from final analysis due to death or discharge before completion of the 14-day observational period, resulting in our final sample of 10 patients.

### 2.1. Data Collection and Biomarker Assessment

Daily assessments included bedside testing of PSP as well as laboratory measurement of CRP, PCT, IL-6, and leukocyte count over a period of 14 days after admission. All biomarkers were measured as part of the same blood draw. In addition, daily calculation of SIRS and NEWS2 was performed.

PSP was determined once a day by using a bedside point-of-care immunoassay (abioSCOPE^®^, Abionic SA, Epalinges, Switzerland) from 50 µL of venous or arterial whole blood. Disposable test kits (IVD CAPSULE PSP) (Abionic SA, Epalinges, Switzerland) were used for the measurements. The blood sample was mixed with fluorescence-labelled detection antibodies in the abioMIX tube and then transferred to the biosensor capsule using a sterile pipette. The quantitative determination of the PSP concentration was performed automatically by the abioSCOPE^®^ device within 7 min. Daily PSP measurements were performed to evaluate temporal biomarker dynamics over the course of intensive care treatment and to assess whether changes in PSP trajectories preceded clinically overt infectious complications. As published in the literature, the quantification range of the immunoassay ranges from 20 to 600 ng/mL. PSP risk categories are defined as follows: <100 ng/mL (low risk), 100–200 ng/mL (moderate risk), 200–300 ng/mL (high risk), and ≥300 ng/mL (very high risk of sepsis).

All diagnostic and therapeutic decisions, including initiation of antimicrobial therapy, were made independently of PSP results. Treating physicians were not provided with PSP measurements during the observation period in order to avoid incorporation bias in clinical decision-making.

### 2.2. Definition of Infectious Events

Diagnosis of pneumonia was established by the treating intensive care team based on a combination of radiographic, clinical, and microbiological findings routinely used in burn intensive care practice. Radiographic criteria included new or progressive pulmonary infiltrates on chest radiography or computed tomography. Clinical criteria included fever, worsening oxygenation, purulent respiratory secretions, and leukocytosis or leukopenia. Bronchoscopic evaluation and microbiological respiratory sampling, including bronchoalveolar lavage or tracheal secretion cultures, were performed when clinically indicated. Due to the exploratory pilot design and limited cohort size, no formal CDC or Sepsis-3 adjudication protocol was prospectively implemented. Bacteremia was defined by positive blood cultures in the presence of clinical signs of infection. Routine wound swabs were collected routinely three times a week and additionally in case of unplanned changing dressings as well as surgeries; however, differentiation between colonization and clinically relevant wound infection was not feasible and therefore not analyzed.

### 2.3. Statistical Analysis

An exploratory statistical analysis was performed. Continuous variables are presented descriptively. Pearson correlation coefficients were calculated to assess associations between PSP, conventional biomarkers, and clinical scores. Correlation strength was interpreted according to Cohen’s criteria. No adjustment for multiple testing was performed.

For further analysis, a linear mixed effect model was fit to data with PSP changes from day 1 at any day from 2 to 7 as dependent variable, PSP day 1 values, day, group (no infection, pneumonia, pneumonia + bacteremia), and day × group interaction as fixed effects, and subject as random effects. The overall effect was assessed by a type II analysis of deviance, reporting the chi-square *p*-value of the interaction term. In addition, pairwise comparisons against the no infection group were performed using Dunnett’s test, with *p*-values adjusted for multiple comparisons. Statistical analyses were performed using R version 4.5.1. www.r-project.org (Welthandelsplatz 1, 1020 Vienna, Austria)

## 3. Results

### 3.1. Patient Characteristics

The cohort consisted of ten consecutive patients admitted to the burn unit. Among them were six males. Patient age ranged from 18 to 66 (median 43) years. The TBSA affected ranged from 11% to 61.5%, with superficial partial-, deep partial- and full-thickness injuries. The ABSI ranged from 4 to 16 (median 6.5). One patient died after completion of the observation period due to multiple organ failure (day 16). Detailed patient characteristics are shown in Table 1.

### 3.2. Infectious Complications

Six patients (60%) developed either radiologically or bronchoscopy proven pneumonia during the observation period. Three patients (30%) developed bacteremia confirmed by positive blood cultures, though for one of the tree patients bacteremia was only confirmed on day 20 outside observation period of 14 days. All three patients with bacteremia experienced concurrent pneumonia. Swabs were positive in 80% of the patients. In addition, seven patients (70%) were administered systemic antibiotics.

### 3.3. Biomarker and Score Dynamics

SIRS criteria were fulfilled in 90% of all patients, typically within the first five days after admission. NEWS2 scores were positive (≥5) in nine of ten patients within the first two days, limiting their discriminatory value.

**Figure 1:**
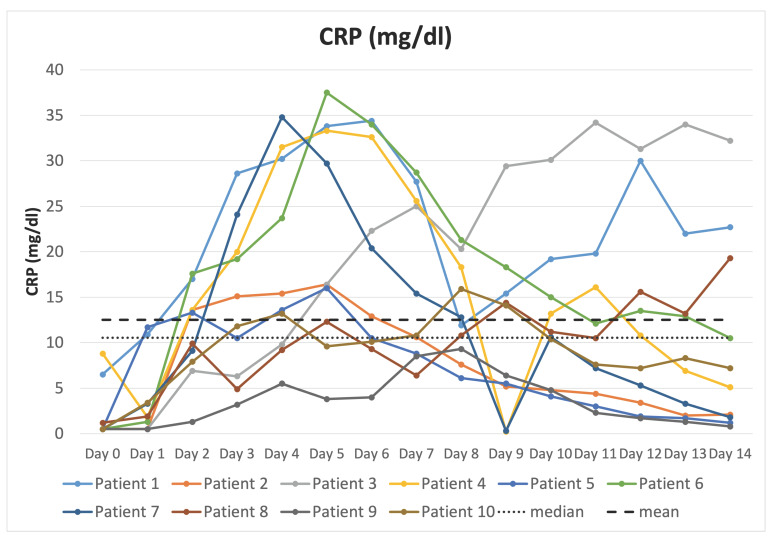
C-reactive protein (CRP) (mg/dL) development over 14 days.

**Figure 2:**
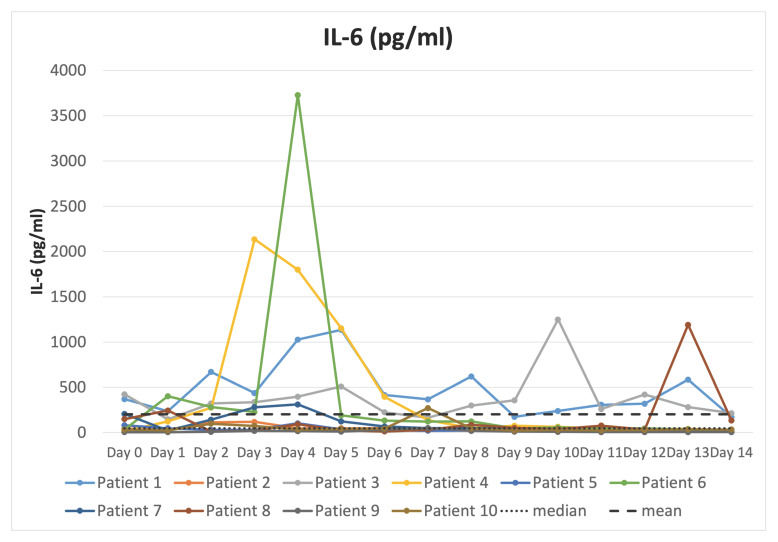
Interleucine-6 (IL-6) (pg/mL) development over 14 days.

**Figure 3:**
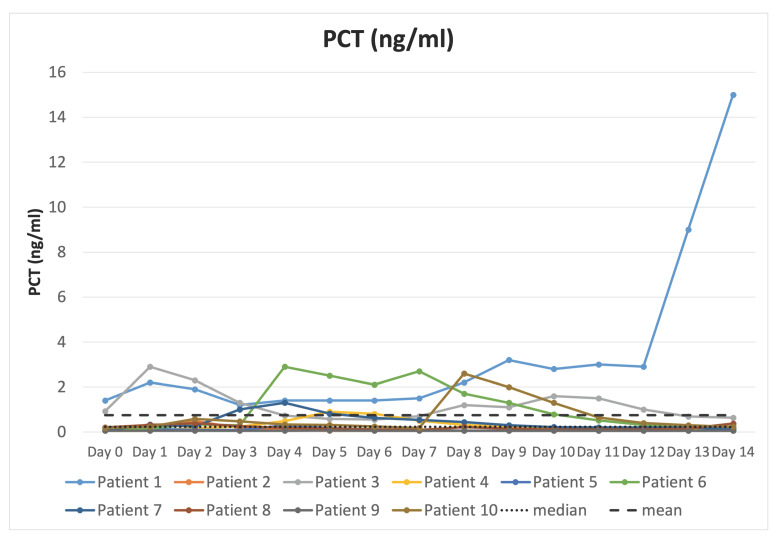
Procalcitonin (PCT) (ng/mL) development over 14 days.

**Figure 4:**
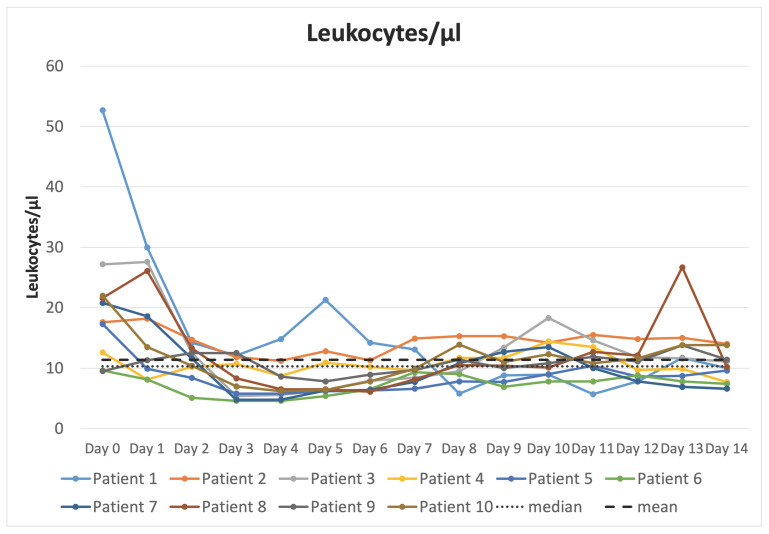
Leukocytes/µL development over 14 days.

Regarding the other biomarkers considered, CRP (Figure 1) and IL-6 (Figure 2) demonstrated early and sustained elevations with pronounced fluctuations. In contrast, PCT (Figure 3) and leukocyte counts (Figure 4) were persistently elevated with limited temporal variability.

Evaluation of PSP curves showed pronounced differences in temporal behavior. In contrast to conventional inflammatory biomarkers, PSP demonstrated comparatively stable and interpretable temporal patterns over the observation period. Distinct trajectory behaviors were observed between patients with and without severe infectious complications, with relatively limited overlap between the identified PSP patterns. Overall, three different trends of PSP trajectories were observed, which were accompanied by differing profiles of infectious complications.

**Figure 5:**
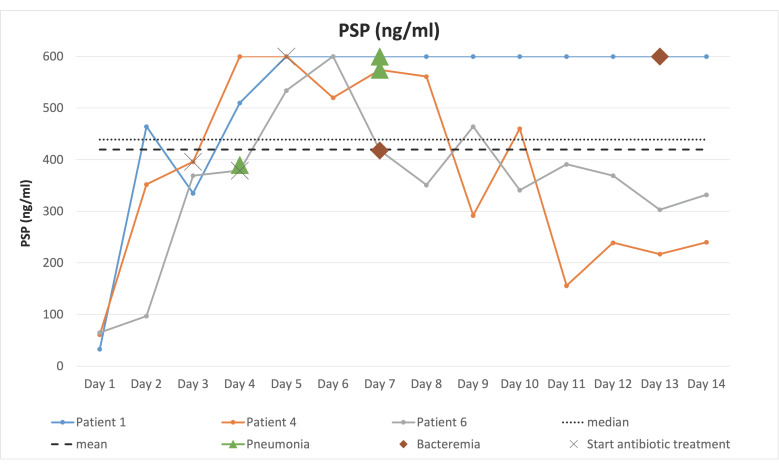
Patients with very high Pancreatic Stone Protein (PSP) levels who developed a pneumonia and bacteremia. Bacteremia for Patient Nr. 2 was only confirmed on day 20 via blood culture and is therefore not marked on the graph.

**Figure 6:**
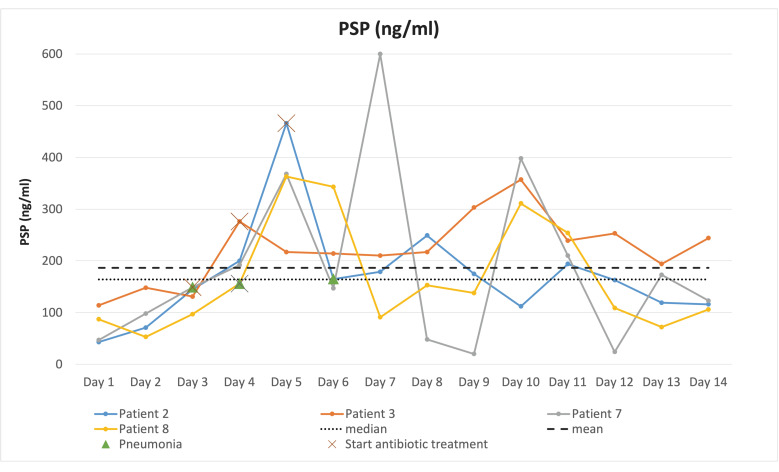
Patients with high Pancreatic Stone Protein (PSP) levels ranging predominantly from 150–450 ng/mL who developed a pneumonia. Patient Nr. 3 developed a pneumonia on day 16 after the 14-day monitoring period and is therefore not included in the six patients with pneumonia.

**Figure 7:**
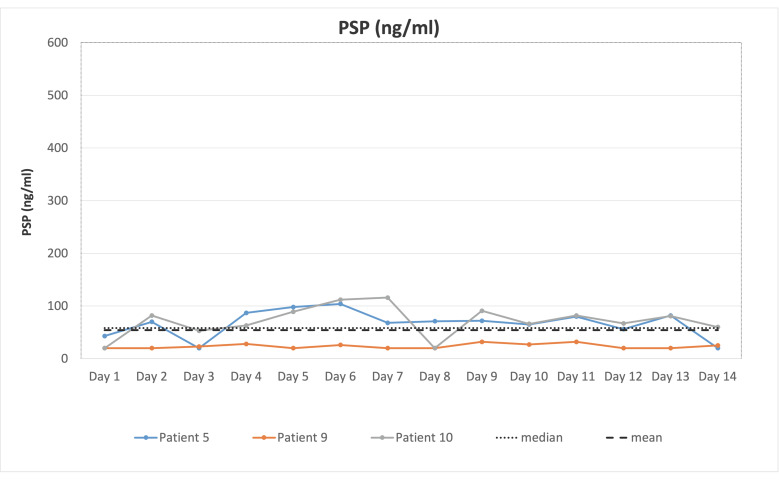
Patients with low levels of Pancreatic Stone Protein (PSP) without a pneumonia or bacteremia.

Three patients (30%) developed both pneumonia and bacteremia; all of them exhibited PSP concentrations exceeding 450 ng/mL (Figure 5). A second subgroup developed pneumonia and showed intermediate PSP levels ranging predominantly from 150 to 450 ng/mL (Figure 6). In contrast, patients in the third subgroup (Figure 7) maintained consistently low PSP concentrations and did not develop pneumonia or bacteremia during the observation period.

In four out six pneumonia cases, PSP rose above 300 ng/mL a mean of 66 h (range 24–120 h) prior to clinical diagnosis. In bacteremia, PSP elevation preceded positive blood cultures by 4 and 18 days.

**Figure 8:**
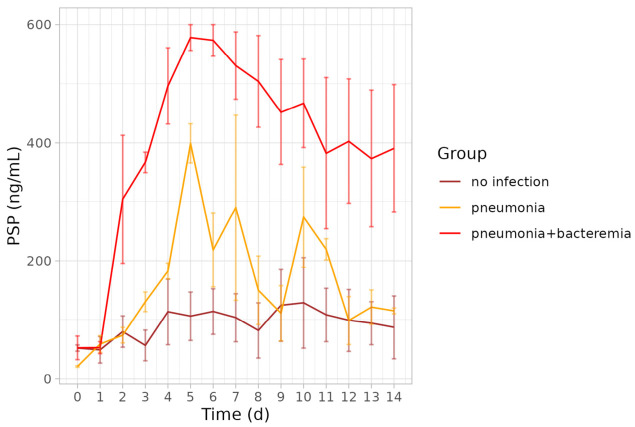
Mean longitudinal pancreatic stone protein (PSP) concentrations over the 14-day observation period stratified by infection status. Error bars represent standard errors of the mean (SEM).

Additional exploratory analysis of longitudinal PSP trajectories demonstrated differing temporal PSP profiles between the clinical subgroups over the observation period (Figure 8). Across all measurement time points, mean PSP concentrations (SD) were 93.3 (82.4) ng/mL in patients without infection, 164.2 (126.3) ng/mL in patients with pneumonia, and 395.1 (192.8) ng/mL in patients with combined pneumonia and bacteremia. Similar differences were observed during the first 7 days after admission, with mean PSP concentrations (SD) of 84.4 (67.1) ng/mL, 171.8 (151.3) ng/mL, and 369.4 (220.9) ng/mL, respectively.

To further explore temporal differences between groups during the first 7 days after admission, an exploratory linear mixed-effects model was performed. Despite the very small group sizes, the overall interaction between time and infection group reached statistical significance (type II analysis of deviance: *p* = 0.0164), suggesting differing PSP trajectories over time between groups. Pairwise comparisons adjusted for multiple testing using Dunnett’s test did not reach formal statistical significance.

**Figure 9:**
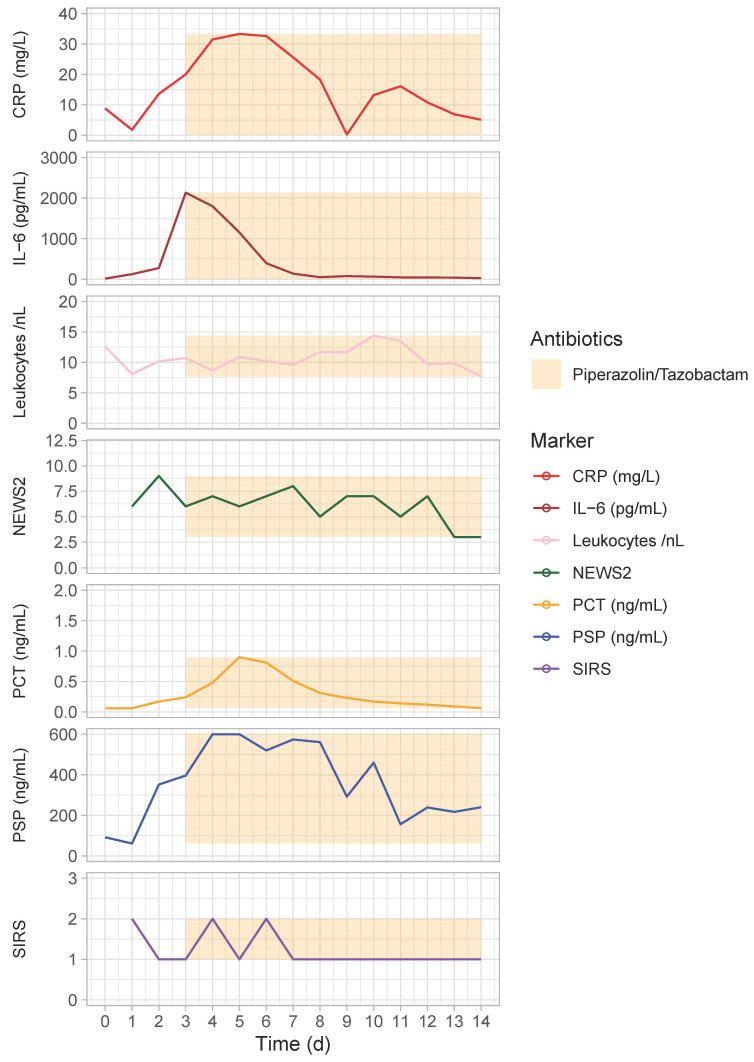
Biomarkers and their development after initiation of antibiotic treatment in a male 32-year-old patient with a 2b degree burn of a Total Body Surface Area (TBSA) of 15%.

Following initiation of antibiotic therapy, declining PSP concentrations were observed in six out of seven treated patients during the subsequent observation period (Figure 9).

In the context of the statistical evaluation of biomarkers using pairwise Pearson correlation shown in Table 2, PSP correlated moderately to strongly with CRP (r = 0.69) and NEWS2 (r = 0.61), moderately with PCT (r = 0.46), and weakly with SIRS (r = 0.23).

## 4. Discussion

This prospective pilot study may indicate that daily bedside PSP measurements may help to identify severe infections rapidly in burn intensive care unit patients at an early stage. In line with the findings of Klein et al. [12,13], our study showed PSP was less affected by burn-related sterile inflammation compared with conventional markers.

The present pilot study observed differing temporal PSP trajectory patterns associated with infectious outcomes in severely burned patients. In this context, our observations suggest differing PSP ranges associated with infectious complications to support the differentiation of infectious states in burn patients. In contrast to the literature, this study identified only three levels of PSP values [13]. In this exploratory cohort, PSP concentrations above approximately 450 ng/mL were predominantly observed in patients with severe infectious complications (bacteremia and pneumonia), between 150 and 450 ng/mL in less severe infections (pneumonia and/or positive wound swabs), and below 150 ng/mL in the absence of bacteremia or pneumonia, consistent with sterile post-burn inflammation. Rather than isolated biomarker concentrations, longitudinal PSP behavior showed differing temporal patterns in patients with and without infectious complications.

Regarding the other biomarkers analyzed and according to the literature, both CRP and IL-6 demonstrated early and pronounced post-traumatic elevations following severe burn injury, accompanied by marked fluctuations over time. Similar patterns have been described by Jeschke et al., who reported sustained inflammatory responses after major burns that were largely independent of infectious complications [4,14,15]. The strong correlation of IL-6 in the early onset of positive blood cultures and an AUC of 69.9% could not be confirmed in our study [6]. As highlighted by Gabay and Kushner, CRP is a nonspecific acute-phase reactant whose serum concentrations may be influenced by a wide range of non-infectious stimuli, including trauma, surgical interventions, or blood transfusions, thereby limiting its utility for infection surveillance [16].

PCT has been extensively studied as a biomarker for sepsis in burn patients. While a meta-analysis by Cabral et al. [17] from 2016 suggested good overall diagnostic performance of PCT, a more recent burn-specific meta-analysis by Chen et al. (2021) [18] reported only moderate diagnostic accuracy in adult burn patients, with substantial heterogeneity across studies. In addition to heterogeneity of the studies, trauma- and surgery-related elevations as well as variable cut-off values were identified as major limitations, especially in the early post-burn period.

In contrast, PSP demonstrated more consistent temporal courses in our pilot study and appeared less susceptible to sterile post-traumatic inflammation, supporting its potential role as an early sepsis biomarker in severe burned patients.

While Klein et al. [13] established diagnostic associations between PSP and sepsis in larger cohorts, our study complements these findings first time by focusing on bedside interpretability of PSP trends in relation to pneumonia or bacteremia development and therapeutic response. In addition, we evaluated bedside PSP measurements using a point-of-care immunoassay (abioSCOPE^®^, Abionic SA, Epalinges, Switzerland) from 50 µL of venous or arterial whole blood. This may offer practical advantages in terms of handling and the timely availability of information, also with regard to the rapid initiation of antibiotic therapy. Delays caused by laboratory analysis can thus be ruled out. This may be particularly relevant in burn intensive care settings, where rapid clinical deterioration can occur and therapeutic decisions often need to be made within short time intervals. The feasibility of bedside PSP assessment may therefore support real-time clinical evaluation and integration into routine intensive care workflows.

The extended lead time observed prior to microbiologically confirmed bacteremia should be interpreted with caution, given the small number of septic events and the time required for blood culture incubation. In this context, early PSP elevation likely reflects ongoing infectious processes preceding microbiological confirmation rather than true diagnostic anticipation of bloodstream infection.

In this study a temporal decline in PSP concentrations was observed in several patients following initiation of antimicrobial therapy. However, given the exploratory design and absence of standardized therapeutic response assessment, these observations must be interpreted cautiously. Alternative explanations including natural clinical recovery, surgical source control, regression to the mean and survivor bias cannot be excluded. Therefore, the present findings do not allow conclusions regarding the utility of PSP for therapeutic monitoring but may support further investigation in larger prospective studies.

Recent studies suggests that biomarker-guided antimicrobial stewardship strategies may reduce unnecessary antibiotic exposure in critically ill patients while maintaining clinical safety. A recent meta-analysis by Papp et al. including more than 9000 ICU patients demonstrated that procalcitonin-guided antimicrobial therapy was associated with shorter antibiotic treatment duration and lower mortality compared with standard care [19].

Similarly, Kubo et al. reported in a recent systematic review that longitudinal biomarker-guided antimicrobial discontinuation strategies may safely reduce antibiotic exposure in septic critically ill patients without increasing mortality risk [20]. However, these studies were not performed for burn patients. In this context, our observations regarding longitudinal PSP trajectory patterns may be clinically relevant in the future. In contrast to CRP, IL-6, and PCT, PSP demonstrated comparatively stable temporal behavior in non-infected burn patients despite extensive tissue injury. At the same time, rising PSP concentrations preceded clinically overt infectious complications in several patients and frequently declined following initiation of antimicrobial therapy. These findings may support the hypothesis that longitudinal PSP assessment could potentially contribute to more individualized antimicrobial decision-making in burn intensive care medicine, particularly in clinical situations where differentiation between sterile post-burn inflammation and true infection remains difficult. Importantly, recent reviews have emphasized that dynamic biomarker trajectories may provide greater clinical utility than isolated single measurements in complex inflammatory ICU populations [21]. However, PSP-guided antimicrobial stewardship has not yet been evaluated in interventional burn studies, and the present exploratory pilot study was neither powered nor designed to assess antibiotic stewardship outcomes, duration of therapy, or resistance development. Therefore, conclusions regarding PSP-guided optimization of antimicrobial therapy cannot currently be drawn and require prospective multicenter validation.

In line with the findings of Gimenez et al. our study showed limited discriminatory value of SIRS criteria in burn populations, where physiological responses to extensive tissue injury, surgical interventions, and intensive care frequently result in false-positive scores [22]. The same applied to NEWS2 in the patient population studied. These findings further underline the need for biomarkers that are less affected by non-infectious inflammatory reactions especially in burn intensive care settings.

Main limitations of this pilot study include the small, single-center sample and exploratory design, limiting generalizability and preventing robust diagnostic accuracy analyses. In addition, five patients were excluded because of early death or discharge before completion of follow-up. This may have introduced selection and survivor bias, as critically ill burn patients with early deterioration could potentially demonstrate differing PSP dynamics compared with patients surviving long enough for longitudinal assessment. Following the exploratory pilot design and limited cohort size, no formal CDC or Sepsis-3 adjudication protocol was prospectively implemented in diagnosis of pneumonia. Correlations were descriptive, and the low number of infectious complications precluded reliable sensitivity, specificity, and ROC analyses. Infections were defined by clinical, radiological, and microbiological criteria without a standardized sepsis definition, introducing potential variability. Additionally, PSP measurements once a day may have limited temporal resolution.

## 5. Conclusions

This prospective pilot study in critically ill burn patients suggests that PSP is a promising early biomarker of severe infection and may complement existing laboratory markers and clinical scores. Longitudinal (trajectory-based) assessment provided clinically relevant differentiation between sterile inflammation and infection, indicating that dynamic monitoring may improve early detection. The findings further indicate that PSP levels may support the stratification of infectious versus non-infectious conditions in this population. Bedside point-of-care testing enables rapid results and may support timely antibiotic management. Integration of PSP trajectory assessment into multimodal diagnostic approaches may further improve infection surveillance in critically ill burn patients. Further large, multicenter studies are needed to confirm diagnostic accuracy, establish optimal cut-offs, and define PSP’s role in guiding antimicrobial strategies.

## Figures and Tables

**Table 1:** Patient characteristics and clinical outcomes. Data are presented for all patients, patients without infection, and patients with infection (pneumonia and/or bacteremia).

	All Patients	Patients with No Infection	Patients with Infection (Pneumonia and/or Bacteremia)
number of patients	10	4	6
male	8	3	5
female	2	1	1
age median	43.0	43.0	42.0
age mean	43.8	41.5	45.
Total Body Surface Area (TBSA) (%), median	25.3	22.3	28.5
Total Body Surface Area (TBSA) (%), mean	27.9	21.4	32.3
inhalation injury	2	1	1
ABSI Score median	6.5	6.0	7.5
ABSI Score mean	7.5	6.0	8.5
length of stay ICU (days), median	16.0	11.5	16.0
length of stay ICU (days), mean	19.4	22.5	17.3
length of stay total (days), median	26.5	34.0	26.5
length of stay total (days), mean	30.9	34.0	28.8
mortality	1	0	1
pneumonia	6	0	6
bacteremia	3	0	3
invasive ventilation (days), median	7.0	2.5	11.0
invasive ventilation (days), mean	7.2	3.0	10.0
number of operations, median	4.00	4.5	4.0
number of operations, mean	4.40	4.5	4.3

**Table 2:** Pearson correlation coefficients (r) are displayed within the matrix and color-coded according to magnitude. Correlation strength was interpreted according to Cohen’s criteria (r = 0.10–0.29 weak, 0.30–0.49 moderate, 0.50–0.69 moderate-to-strong, ≥0.70 strong).

	PCT	IL-6	NEWS2	PSP	CRP	Leukocytes	SIRS
PCT	1.0	0.21	0.45	0.46	0.36	0.02	0.02
IL-6	0.21	1.0	0.38	0.37	0.42	0.05	0.13
NEWS2	0.45	0.38	1.0	0.61	0.57	−0.03	0.23
PSP	0.46	0.37	0.61	1.0	0.69	−0.19	0.23
CRO	0.36	0.42	0.57	0.69	1.0	−0.19	0.17
Leukocytes	0.02	0.05	−0.03	−0.19	−0.19	1.0	0.03
SIRS	0.02	0.13	0.23	0.23	0.17	0.03	1.0

## Data Availability

The data presented in this study are available on reasonable request from the corresponding author due to privacy and ethical restrictions.
